# 4230 An App a Day: Examining Clinical Evidence for Safety and Efficacy of Diabetes Mobile Health Apps

**DOI:** 10.1017/cts.2020.191

**Published:** 2020-07-29

**Authors:** Avantika Pathak, Susan Bain, Eunjoo Pacifici

**Affiliations:** 1 USC School of Pharmacy; 2 USC Department of Regulatory and Quality Sciences

## Abstract

OBJECTIVES/GOALS: Mobile health applications are widely used by the public but vary in how they are classified and regulated. This study examines the evidence of the safety and efficacy of mobile medical applications specifically focusing on those that are used to manage diabetes. METHODS/STUDY POPULATION: To understand the current regulatory landscape of mobile health applications (mHealth apps) for diabetes, a literature survey was conducted using the Pubmed database. Top mHealth apps were identified by searching the Apple store website using 10 key terms associated with diabetes management applications. A maximum of ten results, when available for each key term, were studied by exploring the FDA databases to understand how the products were regulated and if any were subject to recalls. These selected mHealth apps were also searched on clinicaltrials.gov to see if there were ongoing or completed clinical trials and if the trials were designed to include efficacy and safety outcome measures. RESULTS/ANTICIPATED RESULTS: Of the 71 mHealth apps for diabetes management that were identified, 16 were regulated. These products spanned a diverse range of functions including device data and decision support systems. Although 11 had clinical trial data demonstrating efficacy, only 4 had data demonstrating both efficacy and safety. Two of the regulated applications were subject to product recalls due to programming errors that resulted in incorrect insulin dose recommendations. These two applications had clinical trials evaluating efficacy but not safety. The companies noted that the incorrect insulin calculation from their respective mHealth app could cause either a low- or high-impact hypoglycemic event. DISCUSSION/SIGNIFICANCE OF IMPACT: With little to no clinical trial data to support their safety and efficacy, mHealth apps in the diabetes marketplace pose risks for patients as evidenced by recent safety-related recalls. The results of this study indicate that these products may need to be more tightly regulated.

